# MARCH1 as a novel immune-related prognostic biomarker that shapes an inflamed tumor microenvironment in lung adenocarcinoma

**DOI:** 10.3389/fonc.2022.1008753

**Published:** 2022-10-13

**Authors:** Zhiyong Xu, Jun Liu, Zichuan Liu, Haibo Zhang

**Affiliations:** ^1^ Department of Oncology, the Second Clinical Medical School, Guangzhou University of Chinese Medicine, Guangzhou, China; ^2^ Department of Radiotherapy, Southern Theater General Hospital, Guangzhou, China; ^3^ Department of Pulmonary and Critical Care Medicine, The Second Affiliated Hospital of South China University of Technology, Guangzhou, China; ^4^ Internal Medicine Section2, Affiliated Cancer Hospital and Institute of Guangzhou Medical University, Guangzhou, China

**Keywords:** MARCH1, LUAD, E3s, immunotherapy, pan-cancer

## Abstract

E3 ubiquitin ligases (E3s), the second most common cancer-related functional protein family, play vital roles in multiple tumors. However, their importance in prognosis and immunotherapy of lung adenocarcinoma (LUAD) is not clear. First, utilizing the data from The Cancer Genome Atlas (TCGA), we comprehensively assessed the expression profile and immunological association of 13 E3s in LUAD patients. Consequently, MARCH1 was considered a candidate for further study. Second, several algorithms were applied to assess the correlation between MARCH1 and immunological characteristics in the LUAD tumor microenvironment. Third, an immune risk score (IRS) was developed to predict the prognosis. Finally, the immunological relationship of MARCH1 in pan-cancer was also estimated. We found that E3s were disordered in LUAD. Among them, MARCH1 was positively correlated with most immunological characteristics, indicating that MARCH1 designed an inflamed TME in LUAD. Coincidently, LUAD with low MARCH1 expression had a poor prognosis and was not sensitive to immune checkpoint blockers. In addition, the IRS could accurately predict the prognosis. In pan-cancer, MARCH1 was also positively correlated with most immunological characteristics. In conclusion, MARCH1 could be a novel and promising biomarker for immune status and effectiveness of immunotherapy for LUAD patients.

## Introduction

Lung adenocarcinoma (LUAD) is the dominating pathological subtype of lung cancer, which is the deadliest and second most prevalent cancer. Its incidence is still increasing worldwide (1). Moreover, the therapeutic outcome is far from satisfactory due to delayed diagnosis and limitation of traditional treatments.

Tumor cells could be identified as abnormal substances and eliminated by immune cells. Meanwhile, they have special mechanisms to evade host immune surveillance (2). Immunotherapy, with fewer off-target effects and longer-lasting responses, could restore the patient’s immune system to kill tumor cells through natural mechanisms and is rapidly becoming a focus of oncology research (3). Recent cancer treatment applications of immunotherapy include chimeric antigen receptor T cells, vaccine therapy, and immune checkpoint blockers (ICBs) targeting programmed cell death-ligand 1/programmed death protein 1 (PD-L1/PD-1) and cytotoxic T lymphocyte-associated protein 4 (CTLA-4) (4). In clinical application, the US Food and Drug Administration (FDA) has approved several ICBs to treat non-small cell lung cancer (NSCLC), melanoma, and some other malignant tumors (5). Despite these encouraging results, immunotherapy is only effective for a minority. Accumulated evidence revealed that sensitivity to ICBs was strongly related to tumor immune phenotypes, which were classified as inflamed/infiltrated, immune-excluded, and immune-desert phenotypes based on the T cells’ spatial distribution in the tumor microenvironment (TME) (6). An inflamed TME always made immunotherapy more effective than the other two phenotypes. It was characterized by a high PD-L1 and PD-1 expression and a high prevalence of tumor-infiltrating immune cells (TIICs) (7). Consequently, the amount of TIILs and factors regulating the immune cell infiltration, such as cytokines, chemokines, and other components, is crucial for immunotherapy. Meanwhile, elements of inflamed tumors included microsatellite instability (MSI) and tumor mutational burden (TMB) (6, 8). Taken together, these immunologic characteristics within the TME were vital to immunotherapy. Therefore, a biomarker indicating the status of the TME could predict the immunotherapy response.

Ubiquitination, one of the posttranslational modifications, is a cascade that regulates protein degradation by ligating ubiquitin to the target protein. Ubiquitin is activated by binding to ubiquitin-activating enzymes (E1s), subsequently transmitted to ubiquitin-conjugating enzymes (E2s), and finally covalently ligated to a target protein regulated by ubiquitin ligases (E3s) (9). Ubiquitination is an essential system that regulates the stability of numerous pivotal regulatory factors and cellular processes, covering cell cycle, proliferation, apoptosis, and neurotransmission (10). It has been observed to be dysregulated in many cancers (11).

E3s, of which there are about 1,000 members in *Homo sapiens*, can be divided into four categories according to their functional domains: HECT domain-containing type, PHD-finger type, U-box type, and RING-finger type proteins (12). Because of their specificity for substrates, E3s are key regulators in the ubiquitination process. Several immune processes have been linked to their regulation, including immune evasion and antigen presentation, T cell-mediated tolerance, and lymphocyte activation and differentiation (13). Furthermore, ubiquitination of PD-1/PD-L1 *via* E3s seriously alters the protein stabilization and dynamics of PD-1/PD-L1 in cancer immunotherapy (14). However, the relationship between E3s and immunologic signatures in the TME as well as their predictive value in prognosis and immunotherapy efficacy in LUAD remains unknown.

Herein, we obtained 13 E3s, of which the significance in immunity has been uncovered, and demonstrated the relationship between the 13 E3s and immunologic characteristics in the TME. Of interest, MARCH1 was found to have a strong association with the TME. To gain sufficient insight into the role of MARCH1 in LUAD and pan-cancer, we conducted a comprehensive analysis on multiple levels containing mRNA expression, immune signature, patient survival, and chemical compounds. We also established a risk model to predict prognosis and immunotherapy response. Collectively, our systematic analysis provides a comprehensive insight on the biology of MARCH1, which has greater potential value on immunotherapy targets than other E3s.

## Materials and methods

### Data acquisition

All data, including the pan-cancer RNA sequencing data, somatic mutation data, and detailed clinical data, were acquired from The Cancer Genome Atlas (TCGA) database using UCSC Xena. TMB was calculated with somatic mutation data. MSI data were collected from the study of Bonneville et al. (15).

### Expression profiles of E3 ligases

First, the expression profiles of the 13 E3s in tumor tissues and paracarcinoma tissues from LUAD patients were analyzed using the RNA sequencing data. Then, in pan-cancer, differences in MARCH1 level between tumor and paracarcinoma tissues were computed *via* “limma” R package (false discovery rate <0.05, |log_2_FC| ≥1).

### Correlation between MARCH1 and the immunological characteristics in the TME

The characteristics contain the expression level of immunomodulators (16), the expression of immune checkpoints, the infiltration level of TIICs, and the cancer immunity cycle’s activity. The activities of these cancer immunity cycle steps were evaluated by single sample gene set enrichment analysis (17). The association between MARCH1 and immune checkpoints, mismatch repair (MMR) protein was analyzed *via* the Spearman correlation coefficients pan-cancer.

### Association between MARCH1 and therapeutic signatures

We summarized the therapeutic signatures from previous studies. Then, their enrichment scores (ESs) were computed *via* the gene set variation analysis R package. The LUAD-linked drug-target genes were filtered out in the DrugBank database. Their levels were compared between low- and high-MARCH1 group.

### Screening of immune-related differentially expressed RNAs

Considering the median of MARCH1 mRNA expression, immune score, and stromal score, the latter two computed *via* the ESTIMATE R package, LUAD cohorts were parted into corresponding low and high groups. Differentially expressed RNAs (DERs) were identified *via* the limma R package. Gene Ontology (GO) and Kyoto Encyclopedia of Genes and Genomes (KEGG) analyses were calculated by ClusterProfiler R package.

### Establishment of an immune risk score

With a ratio of 7:3, TCGA-LUAD patients were separated into training and validation sets. Univariate Cox analysis was executed in the training set to identify the correlation between DERs and survival. Then, the immune risk score (IRS) was developed *via* least absolute shrinkage and selector operation (LASSO)-multivariate Cox regression (IRS = ∑ *βi* ∗ *RNAi* βi: the coefficient of the ‘i’th IRS RNA expression profile). Referring to the median IRS, patients fell into low and high groups, and their overall survival (OS) was compared by the Kaplan–Meier method and the log-rank test. Furthermore, the IRS was validated in the validation set.

### Survival analysis in pan-cancer

To demonstrate the links between MARCH1 expression and OS, survival analysis was carried out in TCGA using the “survival” package in R (18).

### Statistical analysis

All statistical analyses were executed utilizing the R software v4.0.3. Correlation between certain variables was gauged using Pearson coefficients. Statistical significance was computed by the log-rank test and defined as *p* < 0.05.

## Results

### Landscape, prognostic value, and immunological correlation of E3s in LUAD

**Figure 1:**
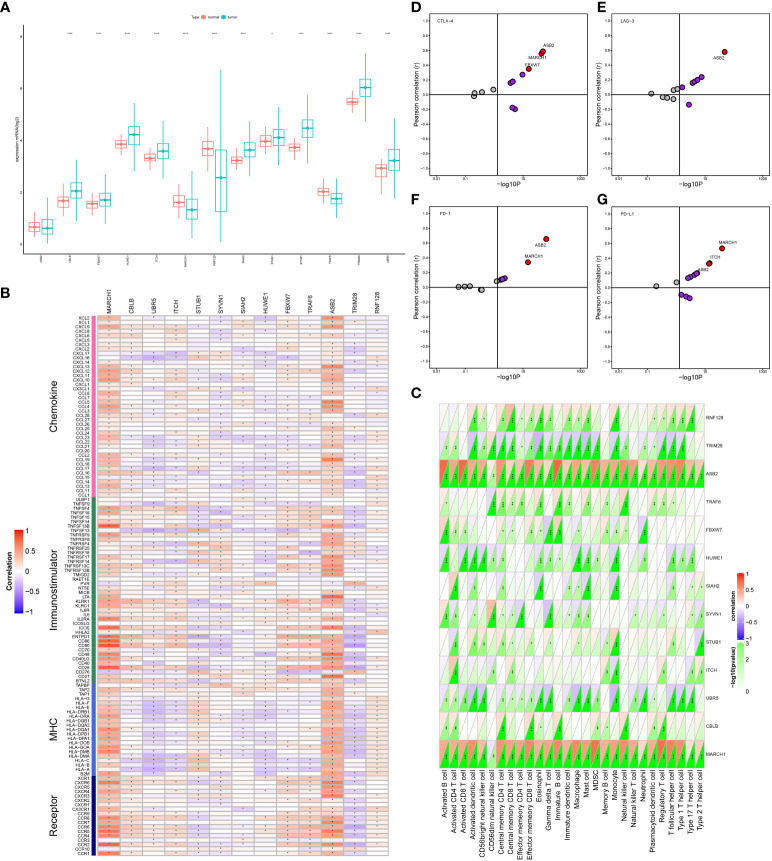
Correlation between E3 ligases (E3s) expression and immunological status in lung adenocarcinoma (LUAD). **(A)** The expression pattern of E3s in tumor and paracarcinoma tissues from TCGA database. **(B)** Relation between E3s and immunomodulators. **(C)** Relation between E3s and tumor-infiltrating immune cells (TIICs). **(D–G)** Relation between E3s and four immune checkpoints. *p < 0.05; **p < 0.01; ***p < 0.001; ****p < 0.0001.

We obtained the expression of the 13 E3s in LUAD from TCGA database. After a comprehensive analysis, we found that the expressions of CBLB, FBXW7, HUWE1, ITCH, SIAH2, STUB1, SYVN1, TRM2B, and UBR5 were significantly upregulated; MARCH1, RNF128, and TRAF6 were significantly downregulated; and ASB2 had no obvious difference between tumor and paracarcinoma (**Figure 1A**).

Our goal was to determine the immunological roles of E3s in LUAD. The results uncovered that E3s had a negative or positive correlation with most immunomodulators and TIICs. Among them, MARCH1 and ASB2 were positively correlated with most immunomodulators and all of the TIICs in this analysis (**Figures 1B, C**). ASB2 expression was correlated with PD-L1, PD-1, CTLA-4, and LAG-3. Simultaneously, MARCH1 expression was correlated with PD-L1, PD-1, and CTLA-4 (**Figures 1D–G**).

These factors, which are crucial for immunotherapy, were positively linked to MARCH1 expression and were more potent than those of other E3s. Moreover, tumor tissue showed a downregulation of MARCH1. We concluded that the downregulation pattern of MARCH1 may be TME specific, indicating the potential of MARCH1 to be a target to improve LUAD immunotherapy. Hence, MARCH1 was regarded as a candidate gene for further study based on its significance in determining prognosis and immune response.

### MARCH1 shapes an inflamed TME in LUAD

**Figure 2:**
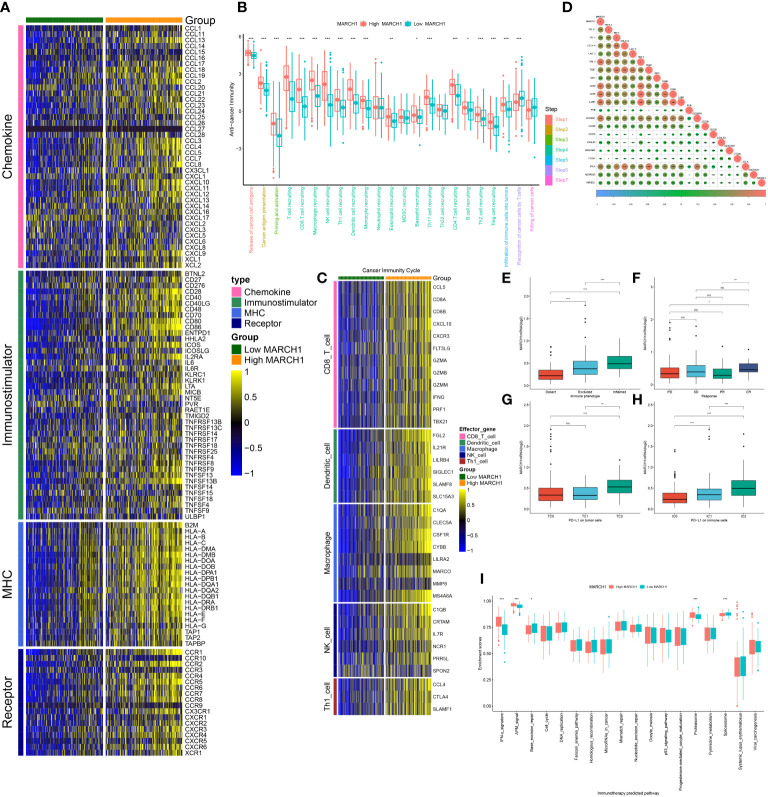
MARCH1 created an infiltrated tumor microenvironment (TME) in LUAD. **(A)** Expression of four types of immunomodulators in low- and high-MARCH1 groups. **(B)** Activity of cancer immunity cycle in low- and high-MARCH1 groups. **(C)** Expression of TIICs’ effector genes in low- and high-MARCH1 groups. **(D)** Relevance between the expression of MARCH1 and immune checkpoints. **(E)** MARCH1 expression in the three immune phenotypes. **(F)** Expression of MARCH1 in four types of clinical outcome of immunotherapy. **(G, H)** Expression of MARCH1 in different cohorts grouped by PD-L1 or PD-1 expression. **(I)** The ESs of pathways for immunotherapy prediction in low- and high-MARCH1 groups. *p < 0.05; **p < 0.01; ***p < 0.001. ns, no significance.

As shown in **Figure 2A**, MARCH1 was positively related to plenty of immunomodulators. Specifically, many major histocompatibility complex molecules (MHCs) were repressed in the low-MARCH1 group. C-X-C motif chemokine ligand (CXCL)9 and CXCL10, two key chemokines promoting the infiltration of CD8+ T, were downregulated in the low-MARCH1 group. In addition, chemokines, such as C-C motif chemokine ligand (CCL)2–5, CCL19, CXCL11, and their corresponding receptors were positively related to MARCH1. In the low-MARCH1 group, activities of most of the steps (Steps 1–5) of the cycle were significantly decreased, indicating a reduced level of TIICs. Of interest, the activities of Steps 6 and 7 were downregulated in the high-MARCH1 group (**Figure 2B**). Furthermore, the infiltration level of TIICs was assessed. As anticipated, MARCH1 had a positive correlation with the effector genes of T helper 1 cells, natural killer cells, macrophages, dendritic cells, and CD8+ T cells (**Figure 2C**). The results also showed that MARCH1 was positively related to numerous immune checkpoints (**Figure 2D**).

In the IMvigor210 cohort, MARCH1 expression was gradually increased from the desert, excluded, to inflamed tumor immune phenotypes. Moreover, in the groups classified based on PD-L1 (TC0, TC1, TC2) or PD-1 expression (IC0, IC1, IC2), MARCH1 expression was highest in the groups with the highest PD-L1/PD-1 expression (TC2 and IC2, respectively) (**Figures 2E, G, H**). Taken together, MARCH1 was strongly linked with the immune phenotype of the TME.

### MARCH1 predicts the clinical response to ICB and other therapeutic options in LUAD

**Figure 3:**
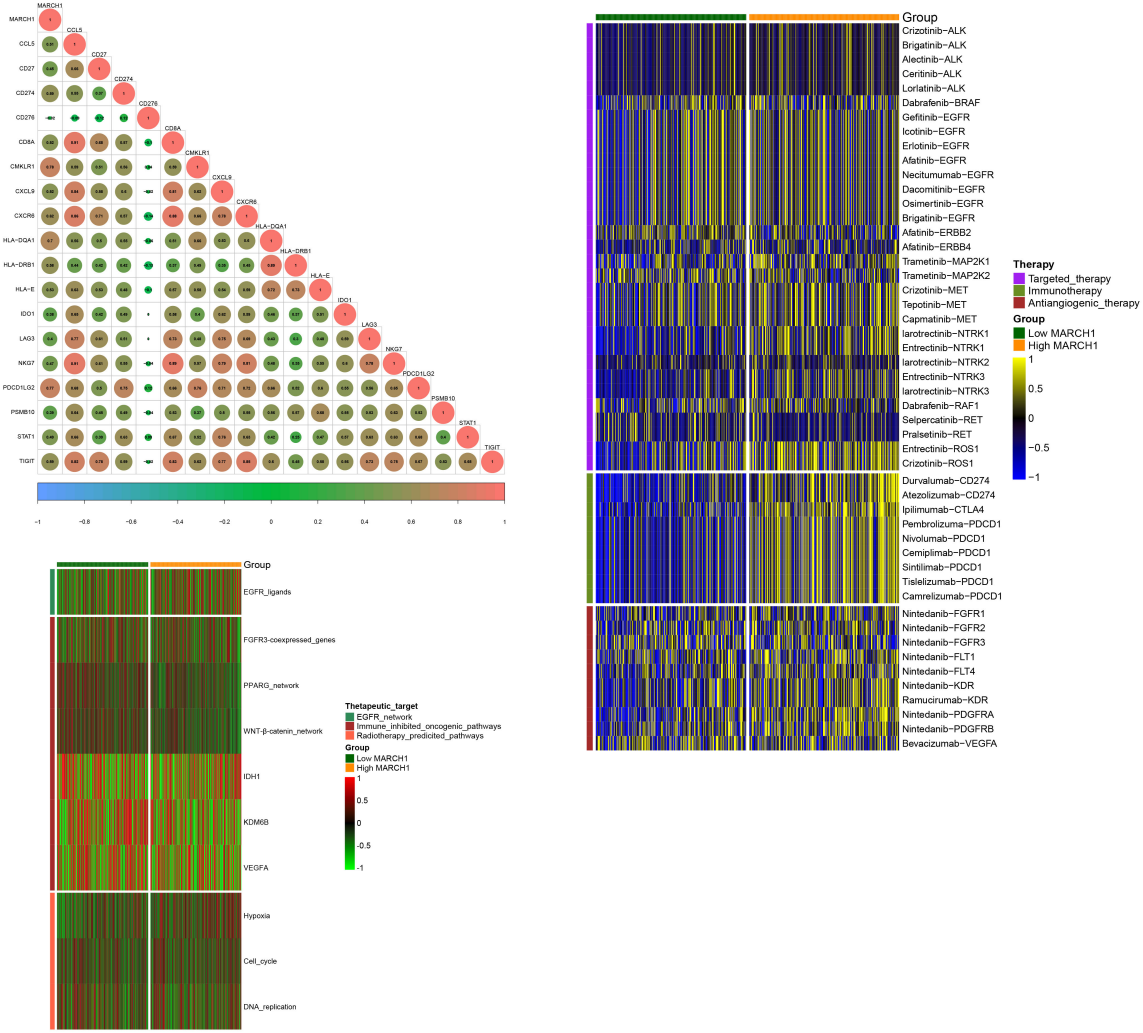
MARCH1 predicts the therapeutic sensitivity in LUAD. **(A)** Relevance between MARCH1 and the T cell inflamed gene signature. **(B)** Relevance between MARCH1 and therapeutic targets. **(C)** Relevance between MARCH1 and LUAD-related drug-target genes.

From the results above, MARCH1 shaped an inflamed TME in LUAD patients, so patients with higher MARCH1 expression ought to be more sensitive to ICBs. Therefore, we further compared the outcome of LUAD patients with distinct MARCH1 expressions. The result showed that MARCH1 expression was significantly higher in patients with complete response to immunotherapy compared to those patients with progressive and stable disease (**Figure 2F**). Positive correlation also existed between MARCH1 and the ESs of three immunotherapy-positive gene signatures: IFN-γ signature, APM signal, and proteasome signal (**Figure 2I**). In addition, MARCH1 had a positive correlation with most individual genes of the T cell inflamed signature (**Figure 3A**). However, there was no discernible difference in the ESs of the therapeutic targets between low- and high-MARCH1 groups, except for the peroxisome proliferator-activated receptor gamma (PPARG) network and WNT-β-catenin network, which were both higher in the former group (**Figure 3B**). Analysis of the association between MARCH1 and drug-targeted genes unveiled an obviously higher sensitivity to specific targeted therapies and immunotherapies in the high-MARCH1 group (**Figure 3C**). In a word, ICB could apply to LUAD patients with a high MARCH1 level but not those with a low MARCH1 level.

### Immune-related DER identification

**Figure 4:**
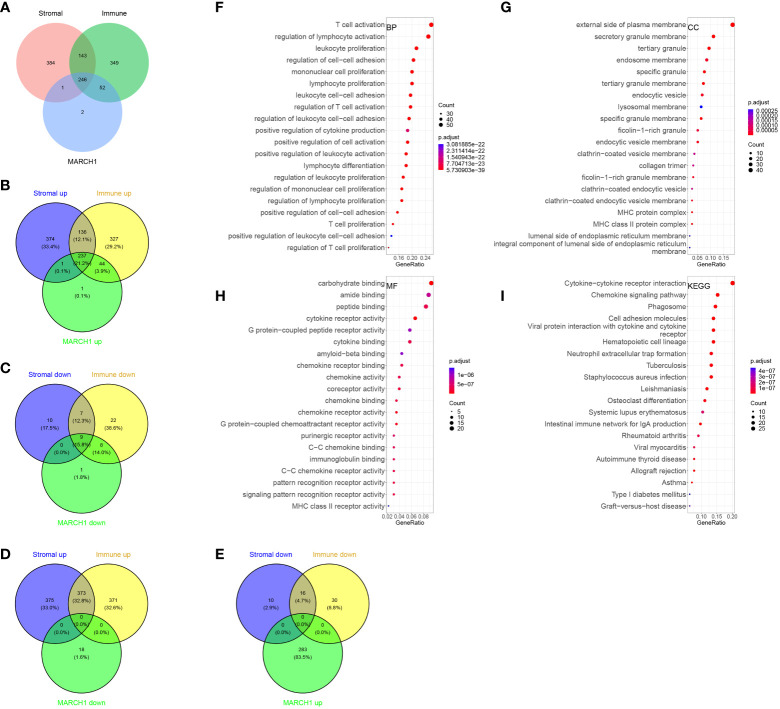
Immune-related differentially expressed RNAs (DERs). **(A–E)** Intersection between DERs in different immune/stromal score groups and different MARCH1 groups. **(F–I)** GO and KEGG analyses of the DERs.

In total, 246 common DERs with prognostic significance were screened out (**Figure 4A**). Notably, there was no overlap among downregulated DERs in the low-MARCH1, high-stromal score, and immune score group. Likewise, no intersection was found among downregulated DERs in the high-MARCH1, low-stromal score, and immune score group (**Figures 4D, E**). It indicated that MARCH1 expression positively related to stromal score and immune scores in the LUAD TME. Furthermore, GO and KEGG analyses revealed that these DERs were involved in immune-related processes (**Figure 4**).

### IRS establishment and validation

**Figure 5:**
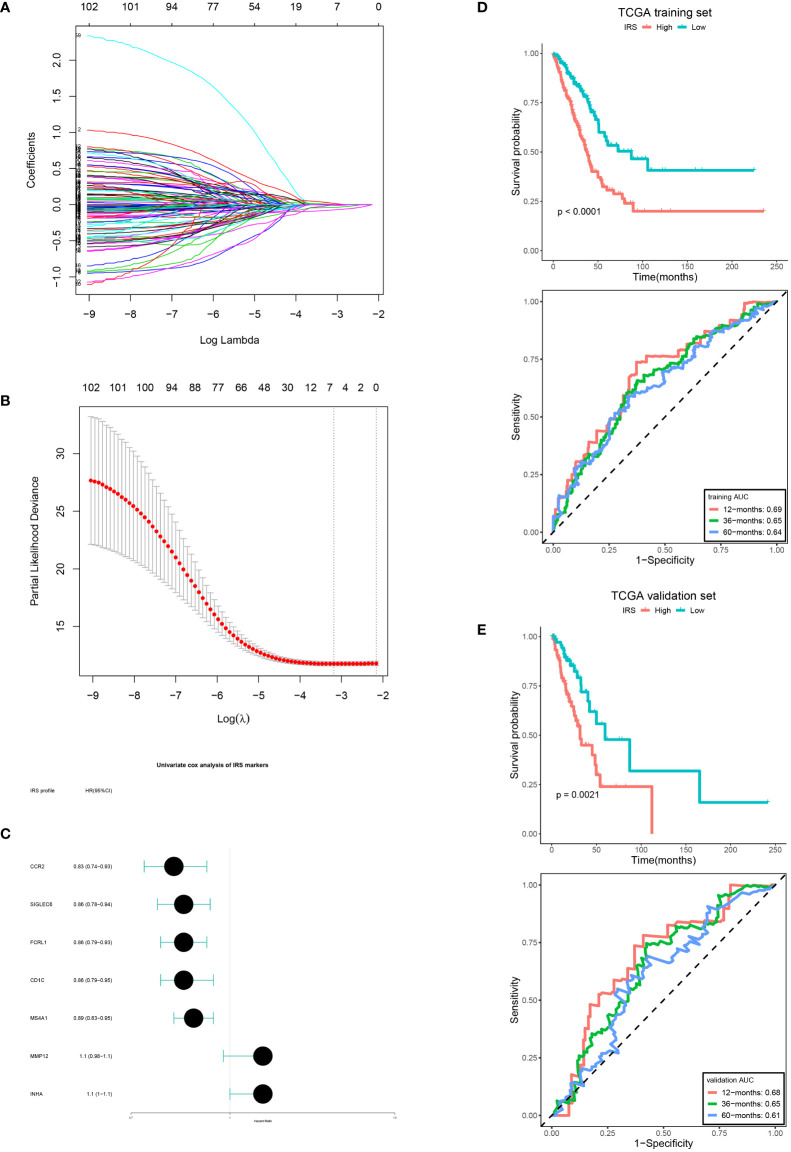
IRS development and verification. **(A, B)** LASSO coefficient of the DERs with prognostic value. **(B)** Cross-validation for turning parameter selection. **(C)** IRS markers displayed as forest plot. OS curves of the low and high IRS and AUCs in the training set **(D, E)** validation set.

According to univariate Cox analysis, 102 DERs had prognostic values. Among them, seven DERs with minimal λ (0.04141) were considered as the best candidates *via* the LASSO algorithm (**Figures 5A–C**). Then, a multivariate Cox regression analysis was performed to develop an IRS according to the seven DERs. Considering the IRS median, 350 patients from TCGA training set were sorted into low- (n = 175) and high-IRS groups (n = 175). The result showed that patients from the low-IRS group had remarkably longer OS than those from the high-IRS group. At 1, 3, and 5 years, the AUCs of the IRS were all more than 0.6 (**Figure 5D**). Furthermore, verification of the prediction accuracy in TCGA validation set displayed that the AUCs of the IRS in the validation and training sets were very similar (**Figure 5E**). Taken together, this model could steadily predict the prognosis.

### MARCH1 expression profiles and the correlation with prognosis in pan-cancers

**Figure 6:**
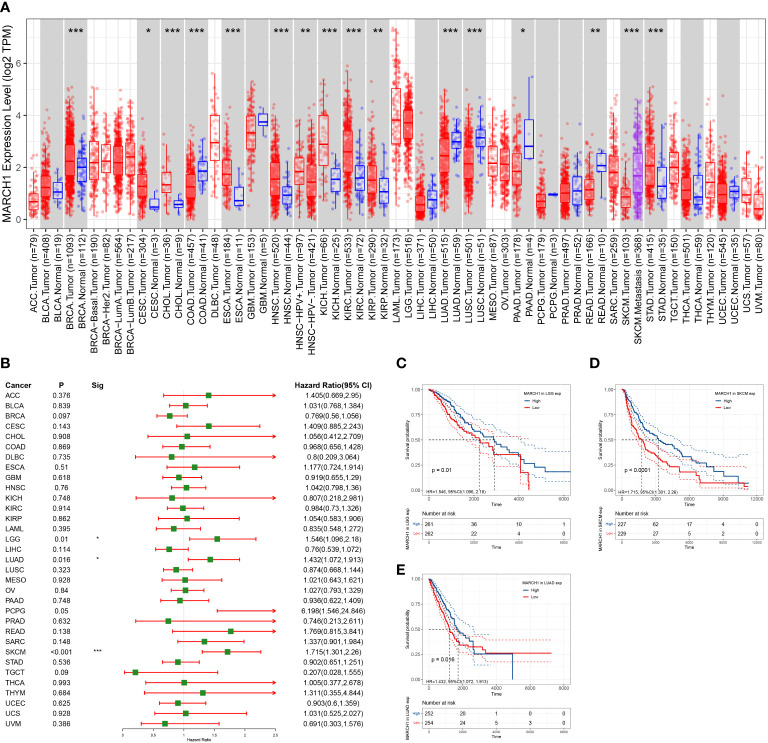
Expression profile and prognosis in pan-cancer. **(A)** MARCH1 expression levels in different types of cancer. **(B)** Relation between MARCH1 expression and prognosis in pan-cancers. **(C–E)** OS curves with significance in three types of cancer (LGG, LUAD, and SKCM). *p < 0.05; **p < 0.01; ***p < 0.001.

To clarify the expression profile of MARCH1 in pan-cancer, MARCH1 levels between tumor and paracarcinoma tissue were compared in 33 cancers. MARCH1 expression was significantly upregulated in breast cancer (BRCA), cervical squamous cell carcinomas (CESC), cholangiocarcinoma (CHOL), esophageal carcinoma (ESCA), head and neck squamous cell carcinoma (HNSC), kidney chromophobe (KICH), kidney renal clear cell carcinoma (KIRC), kidney renal papillary cell carcinoma (KIRP), and stomach adenocarcinoma (STAD). Meanwhile, MARCH1 expression was significantly decreased in colon adenocarcinoma (COAD), LUAD, lung squamous cell carcinoma (LUSC), prostate adenocarcinoma (PAAD), and rectum adenocarcinoma (READ) (**Figure 6A**).

In pan-cancer, the significance of MARCH1 in prognosis was analyzed. The result revealed that a high expression of MARCH1 was always linked with a better OS in lower grade glioma (LGG), LUAD, and skin cutaneous melanoma (SKCM) (**Figure 6B**). OS curves in different cancers showing significant differences between high- and low-MARCH1 groups are exhibited in **Figures 6C–E**.

### Genome-wide relation of MARCH1 expression in pan-cancer

**Figure 7:**
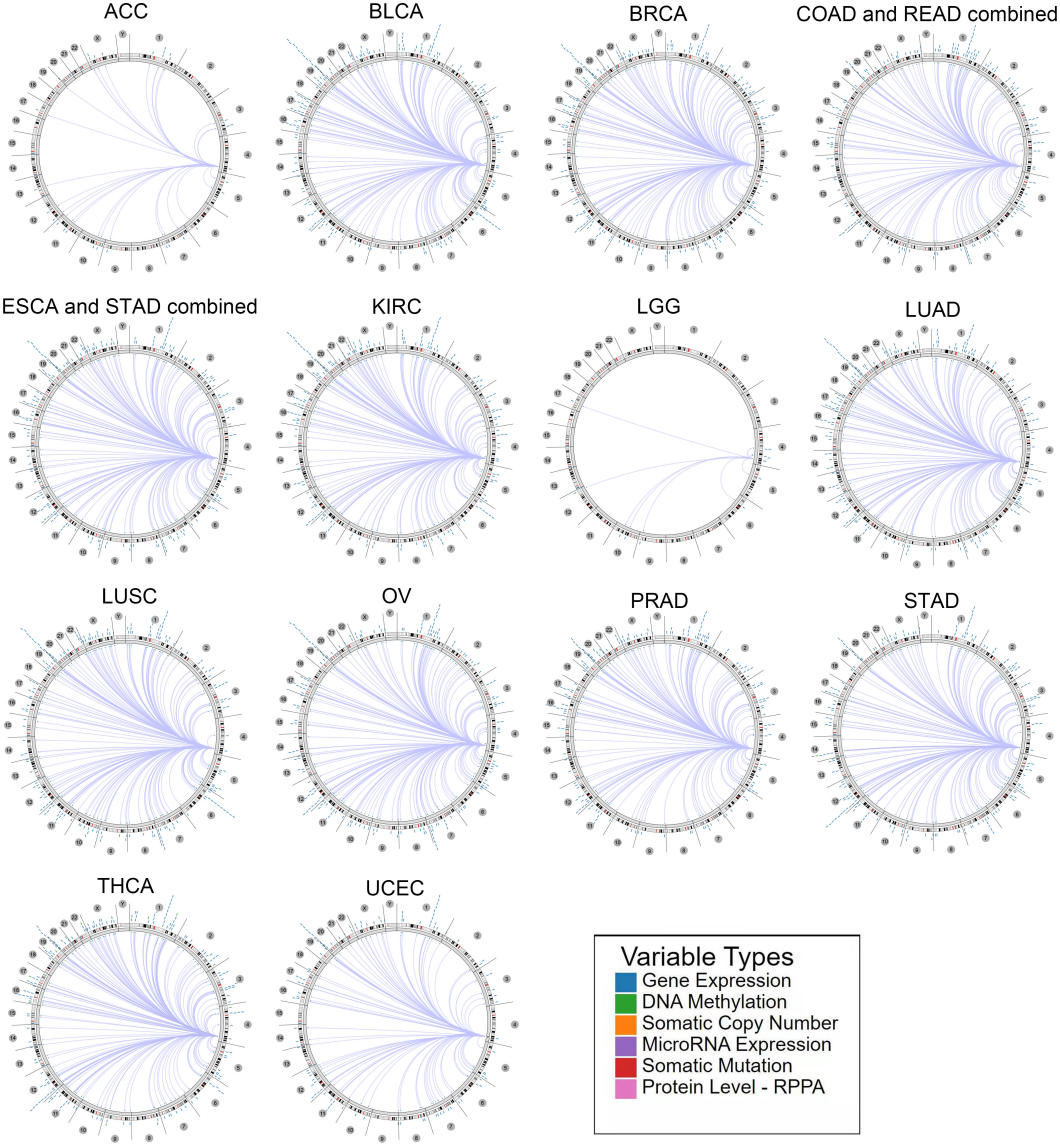
Circus plots displayed the relationship between MARCH1 and other genomic signatures.

The association between MARCH1 and genomic signatures (DNA methylation, somatic copy number, somatic mutation, protein level) was explored *via* the Regulome Explorer web tool. Circus plots illustrated that genome-wide correlations existed in many cancers. **Figure 7** displays the particulars.

### Correlation between MARCH1 and immunological characteristics in pan-cancer

**Figure 8:**
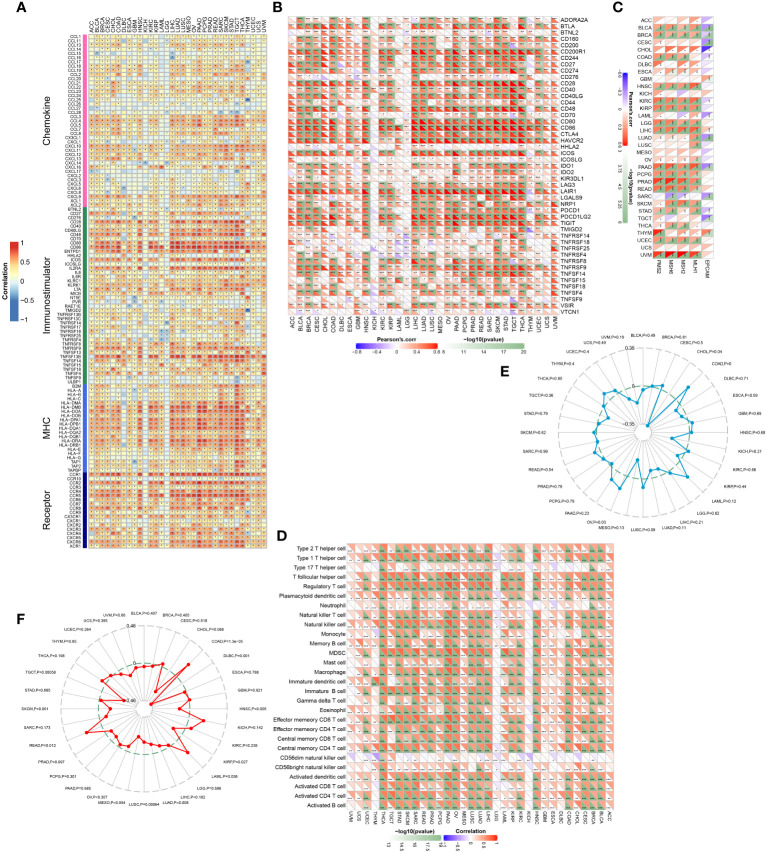
Associations between MARCH1 and immunological status in pan-cancer. Correlation between MARCH1 and **(A)** immunomodulators, **(B)** immune checkpoints, **(C)** MMR signatures, **(D)** TIICs, **(E)** TMB, and **(F)** MSI. *p < 0.05; **p < 0.01; ***p < 0.001.

We analyzed the associations between MARCH1 expression and immunomodulators, the abundance of TIICs in pan-cancer. The result displayed that MARCH1 had a positive correlation with most immunomodulators and TIICs in pan-cancer, except in KICH and LGG (**Figures 8A, D**). Furthermore, we found correlations between MARCH1 expression and confirmed immune checkpoints. MARCH1 was also discovered to have a significant positive correlation with large numbers of immune checkpoints in pan-cancer excluding KICH and LGG (**Figure 8B**). The correlations between MARCH1 expression and five vital MMR signatures, EPCAM, MLH1, MSH2, MutS MSH6, and PMS2, were also detected. The result revealed that MMR signatures, except EPCAM, were positively associated with MARCH1 expression. MARCH1 expression and TMB also had a significant positive association in COAD and ovarian serous cystadenocarcinoma (OV) and a significant negative association in CHOL (**Figure 8E**). MARCH1 expression was non-significantly correlated with MSI in most types of cancer. However, in COAD, acute myeloid leukemia (LAML), and READ, a higher level of MARCH1 meant significantly higher MSI, while in diffuse large B-cell lymphoma (DLBC), KIRP, LUAD, LUSC, SKCM, and testicular germ cell tumors (TGCT), the opposite trend was observed (**Figure 8F**).

## Discussion

Accumulating evidence shows that E3s are strongly related to cancer immunity (19–22). Nonetheless, their value in prognosis and immunological prediction remains unclear. Hence, we selected 13 E3s that have been reported to be related to the immune system to identify a novel and robust marker that could predict the immunotherapy response.

Dysregulation of E3s is frequently observed in numerous cancers and aids tumor cells evading the immune system (13, 22). Consistently, in this study, 12 out of the 13 E3s were significantly upregulated or downregulated in tumor tissue from LUAD patients (**Figure 1A**). This situation further implied that E3s play crucial roles in cancer. Considering their function in cancer immunity, the associations between E3s and immunomodulators, immune cells, and immune checkpoints in the TME were analyzed. The results revealed correlations between E3s and most of these immune-related factors. Among those E3s, MARCH1 expression had a positive correlation with most of the immunomodulators, immune cells, and checkpoints in LUAD and many other cancers (**Figures 1B, C**; **8A, B, D**). Therefore, MARCH1 was regarded as a candidate gene for immunotherapy response prediction.

Accurate prediction of the immunotherapy sensitivity could guide the clinical treatment of cancer. Some recognized biomarkers, such as TMB and MMR defects, have been used to predict immunotherapy sensitivity (23, 24). Previous studies have discovered that MMR is essential for identifying and repairing mismatched bases during DNA replication (25). Therefore, DNA MMR deficiency typically generated high TMB (26) and MSI (27). They contribute to tumor initiation and are independent predictors of ICB efficacy (25). Recent studies concentrated on discovering more precise, convenient, and economical molecular techniques for clinical applications through the development of personalized medicine in a variety of solid tumors. Consequently, there is an urgent need to search for additional biomarkers that can aid clinical immunotherapy. However, there are still no signatures to accurately predict immunotherapy sensitivity.

In this study, we separated the LUAD patients into two groups depending on their level of MARCH1 expression: low- and high-MARCH1 groups. Interestingly, the expression of most immunomodulators, activities of cancer immunity cycle steps, and ESs of some predictable pathways were elevated in the high-MARCH1 group (**Figures 2A, B**; **3D**). The cancer immunity cycle is the procedure of the immune response to tumor cells. The activities of these steps comprehensively determine the antitumor effect of the complicated immunomodulatory interplays in the TME (16). In this study, we discovered that MARCH1 was positively associated with nearly all steps (except killing of cancer cells) of the cancer immunity cycle. Upregulation of immune checkpoints, including PD-L1/PD-1, is also an important characteristic of the inflamed TME, which is triggered by preexisting TIICs in the TME (28). ICBs that target these immune checkpoints have provided LUAD patients with the potential for therapeutic effect and survival. Interestingly, we found that the MARCH1 expression level was positively correlated with the expression of immune checkpoints and TIICs. Moreover, there is relevance between MARCH1 expression level and tumor immunotype. The MARCH1 mRNA level ranged from low to high in desert, excluded, and inflamed immune phenotypes (**Figure 2E**). We also established an IRS for prognosis prediction on the basis of immune-related DERs. Moreover, the IRS model was validated well in the internal validation cohort. In summary, both MARCH1 and IRS may serve as prognostic biomarkers, which robustly illustrate the importance of MARCH1 in prognosis. Furthermore, MARCH1 can also predict the ICB response and define an inflamed TME. High MARCH1 expression always meant that LUAD patients were sensitive to ICBs. However, MARCH1 was negatively correlated with TMB and MSI in LUAD (**Figure 8C**). This contradictory relationship may interpret why TMB and MSI could not always predict the response to ICBs properly. Therefore, we reckoned that the combination of several signatures to predict the sensitivity to ICBs might be a more accurate way. MARCH1 has displayed its powerful modulation in the immune system *via* controlling stability and transforming of some key immunoreceptors, such as the antigen presenting molecule MHC II and costimulatory molecule CD86 (29). Researchers are currently focusing little on the cancer biology of MARCH1 in certain cancers. MARCH1 could inhibit tumor cell growth *in vivo* and *in vitro* in bladder cancer. Meanwhile, ciRs-6 could increase the expression of MARCH1 *via* sponging miR-653 (30). However, Ying Meng et al. discovered that tumor tissue overexpressed MARCH1 relative to paracarcinoma tissues in ovarian cancer (31). Furthermore, the silencing of MARCH1 could restrain the proliferation, migration, and invasion of tumor cells *via* Wnt/β-catenin and nuclear factor-κB pathways (31). Xie L et al. (32, 33) declared that MARCH1 could also provoke tumor progression in hepatocellular carcinoma *via* PI3K-AKT pathway. Collectively, MARCH1 functions differently depending on the type of cancer. This study revealed that MARCH1 was upregulated in some types of cancer and downregulated in others. In most cancers, excluding LGG and KICH, MARCH1 expression was positively associated with most immunomodulators, checkpoints, and infiltrating immune cells (**Figures 8A, B, D**). Therefore, the role of MARCH1 in pan-cancer requires further investigation. In LUAD, we found that MARCH1 expression was positively correlated with the abundance of different kinds of TIICs, including activated CD4+ and CD8+ cells. Activated CD4+ and CD8+ T cells could kill tumor cells. In addition, CXCL9 and CXCL10, two key chemokines, could recruit CD8+ T cells into the TME (16) and were upregulated in the high-MARCH1 group (**Figure 2A**). Collectively, we speculated that MARCH1 may regulate CD8+ T cell recruitment to shape an inflamed TME.

As demonstrated previously, MARCH1 expression is essential for immunotherapy responses. However, MARCH1 is suppressed in LUAD tumor tissue, while the factors that regulate MARCH1 transcription are unknown (34). Therefore, the mechanism by which MARCH1 affects cancer immunity and regulation of MARCH1 expression merit additional research. In addition, LUAD with low MARCH1 expression was insensitive to ICBs. Therefore, it is imperative to seek superior treatment options for LUAD patients expressing low levels of MARCH1.

The research on MARCH1 in cancer immunity is poor. This study firstly demonstrated the role of MARCH1 in prognosis and TME shaping. It also revealed the overall correlation between MARCH1 and immunological characteristics and filled up the gap in this field. MARCH1 is a novelty and robust biomarker to predict the response to immunotherapy and some targeted therapy. It provides a theoretical basis for combined therapy. Additionally, MARCH1 may promote infiltration of CD8+ T cells to shape an inflamed TME and further affect immunotherapy sensitivity. It provides a direction for future research.

There were also limitations in this study. Firstly, clinical and animal studies are necessary to validate the expression profiles of MARCH1 and the correlation between MARCH1 and immunological characteristics. Secondly, the optimal cutoff value for grouping the MARCH1 expression must be determined. Thirdly, more cohorts should be used to validate the results to reduce the batch effects.

## Conclusions

This study demonstrated that MARCH1 could shape an inflamed TME and predict the prognosis and immunotherapy sensitivity in LUAD. Therapies that target its regulator to upregulate the expression of MARCH1 may be an efficient means of improving immunotherapy.

## Data availability statement

The datasets presented in this study can be found in online repositories. The names of the repository/repositories and accession number(s) can be found in the article/supplementary material.

## Author contributions

Conceptualization, ZX and HZ. Data collection, JL. Data analysis, ZX and ZL. Writing, ZX and JL. Revising, ZL and HZ. All authors contributed to the article and approved the submitted version.

## Funding

This study was supported by the Medical Scientific Research Foundation of Guangdong Province, China [No. A2021279].

## Conflict of interest

The authors declare that the research was conducted in the absence of any commercial or financial relationships that could be construed as a potential conflict of interest.

## Publisher’s note

All claims expressed in this article are solely those of the authors and do not necessarily represent those of their affiliated organizations, or those of the publisher, the editors and the reviewers. Any product that may be evaluated in this article, or claim that may be made by its manufacturer, is not guaranteed or endorsed by the publisher.
